# Short and Long-Term Toxicity in Pediatric Cancer Treatment: Central Nervous System Damage

**DOI:** 10.3390/cancers14061540

**Published:** 2022-03-17

**Authors:** Iside Alessi, Anna Maria Caroleo, Luca de Palma, Angela Mastronuzzi, Stefano Pro, Giovanna Stefania Colafati, Alessandra Boni, Nicoletta Della Vecchia, Margherita Velardi, Melania Evangelisti, Alessia Carboni, Andrea Carai, Luciana Vinti, Massimiliano Valeriani, Antonino Reale, Pasquale Parisi, Umberto Raucci

**Affiliations:** 1 Department of Hematology/Oncology, Gene Therapy and Hematopoietic Transplantation, Bambino Gesù Children’s Hospital, IRCCS, 00165 Rome, Italy; annamaria.caroleo@opbg.net (A.M.C.); luciana.vinti@opbg.net (L.V.); 2 Child Neurology Unit, Department of Neuroscience, Bambino Gesù Children’s Hospital, IRCCS, 00165 Rome, Italy; luca.depalma@opbg.net (L.d.P.); stefano.pro@opbg.net (S.P.); massimiliano.valeriani@opbg.net (M.V.); 3 Neuroradiology Unit, Imaging Department, Bambino Gesù Children’s Hospital, IRCCS, 00165 Rome, Italy; gstefania.colafati@opbg.net (G.S.C.); alessia.carboni@opbg.net (A.C.); 4 Department of Maternal Infantile and Urological Sciences, Sapienza University of Rome, 00161 Rome, Italy; alessandra.boni@opbg.net; 5 Department of Emergency, Acceptance and General Pediatrics, Bambino Gesù Children’s Hospital, IRCCS, 00165 Rome, Italy; nicoletta.dellavecchia@opbg.net (N.D.V.); antonino.reale@opbg.net (A.R.); umberto.raucci@opbg.net (U.R.); 6 Child Neurology, NESMOS Department, Faculty of Medicine and Psychology, Sant’Andrea Hospital, Sapienza University of Rome, 00189 Rome, Italy; margherita.velardi@uniroma1.it (M.V.); melania.evangelisti@uniroma1.it (M.E.); pasquale.parisi@uniroma1.it (P.P.); 7 Neurosurgery Unit, Department of Neuroscience, Bambino Gesù Children’s Hospital, IRCCS, 00165 Rome, Italy; andrea.carai@opbg.net

**Keywords:** neurotoxicity, pediatrics cancer, chemotherapy

## Abstract

**Simple Summary:**

The purpose of this review is to describe central nervous system side effects in the treatment of pediatric cancer patients. Unfortunately, we must consider that the scarce data in the literature does not allow us to expand on some issues, especially those related to innovative immunotherapy. We have described the major neurotoxicities arising with the various types of treatment to help specialists who approach these treatments recognize them early, prevent them, and treat them promptly.

**Abstract:**

Neurotoxicity caused by traditional chemotherapy and radiotherapy is well known and widely described. New therapies, such as biologic therapy and immunotherapy, are associated with better outcomes in pediatric patients but are also associated with central and peripheral nervous system side effects. Nevertheless, central nervous system (CNS) toxicity is a significant source of morbidity in the treatment of cancer patients. Some CNS complications appear during treatment while others present months or even years later. Radiation, traditional cytotoxic chemotherapy, and novel biologic and targeted therapies have all been recognized to cause CNS side effects; additionally, the risks of neurotoxicity can increase with combination therapy. Symptoms and complications can be varied such as edema, seizures, fatigue, psychiatric disorders, and venous thromboembolism, all of which can seriously influence the quality of life. Neurologic complications were seen in 33% of children with non-CNS solid malign tumors. The effects on the CNS are disabling and often permanent with limited treatments, thus it is important that clinicians recognize the effects of cancer therapy on the CNS. Knowledge of these conditions can help the practitioner be more vigilant for signs and symptoms of potential neurological complications during the management of pediatric cancers. As early detection and more effective anticancer therapies extend the survival of cancer patients, treatment-related CNS toxicity becomes increasingly vital. This review highlights major neurotoxicities due to pediatric cancer treatments and new therapeutic strategies; CNS primary tumors, the most frequent solid tumors in childhood, are excluded because of their intrinsic neurological morbidity.

## 1. Introduction

Therapy effectiveness in the onco-hematologic field has significantly improved within the past decades, and the childhood cancer survival rate approaches 80% at 5 years from diagnosis. The increased rate of survival is associated with more children who display long-term disabilities and neurological ones in particular. Long-term sequelae of pediatric cancer treatment include auditory, ocular, cardiovascular, pulmonary, gastrointestinal, endocrinological, reproductive, metabolic, and, of course, neurological and neurocognitive [1].

The approach to pediatric oncology patients with neurologic consequences related to toxicity requires a detailed history and examination along with the consideration of multiple, sometimes compounding, etiologies. As treatment options are rapidly expanding and survivorship is increasing, the spectrum of both acute and chronic neurologic symptoms that can be attributed to cancer treatment is also expected to expand [1].

Clinical trials in pediatric oncology continue to be designed with two goals in mind: to improve the cure rates in groups of children where therapy has been less successful and to prevent potential long-term toxicity. Lowering both doses of radiation and drugs that have potential long-term toxicity decreases long term sequelae [2].

Prevention, early recognition, and treatment are essential to ensure the most effective therapeutic regimens for children and to improve the neurological function and quality of life for childhood cancer survivors.

While there are several reports in the literature that review neurologic complications and their prognoses in adults with cancer [2], there remains a relative paucity of literature concerning the neurologic sequelae in the pediatric cancer population. The purpose of this article is to review the literature regarding the central nervous system (CNS) complications of patients treated for cancer in childhood. However, sequelae derived from the CNS location of disease, whether primary or metastatic, will not be addressed because our focus is on the toxic damage caused by traditional as well as newer and emerging treatments. We performed a Medline search using the following keywords: neurotoxicity, pediatric cancer, chemotherapy, radiotherapy, and immunotherapy.

## 2. CNS Effects of Traditional Chemotherapy

Summary statement about chemotherapy: this is the largest session of the paper as the data on chemotherapy in the literature are the most extensive, being the most studied and oldest treatment strategy in this type of patient. To make it clearer, we have created a summary table (Table 1).

Neurotoxicity is a substantial burden of traditional chemotherapy in pediatric cancer treatment. Many chemotherapeutic agents cause CNS side effects, particularly when administered intrathecally or at a high dosage. The most frequent side effects include encephalopathy, cerebellar degeneration, myelopathy, and effects on vision, hearing, and taste [3]. CNS neurotoxicity is also exacerbated by the co-administration of drugs or by combination with radiotherapy [2].

We report the CNS effects of the most widely used chemotherapy drugs in pediatric cancer treatment divided by type (Table 1):

## 3. CNS Radiation Toxicity

Summary statement about radiotherapy: this session is also quite large thanks to

→ the important data in the literature. We have divided the consequences of
→ radiotherapy treatment according to the period of onset by inserting a simplified
→ table and then described in detail the long-term consequences of this
→ type of treatment in pediatric cancer patients.

All forms of ionizing radiation, ranging from nearly weightless photons to particles such as protons or carbon ions, have the potential to produce toxicity in the central nervous system (CNS) [15].

Ionizing radiation can damage tissues through direct and indirect effects, either by directly affecting DNA or by inducing radiolysis of cellular water which generates free radicals that harm DNA and cause metabolic stress to which nerve cells are particularly susceptible [15,16].

The etiology of CNS dysfunction in patients after irradiation is multifactorial [17], influenced by age, comorbidities, psychological and genetic predispositions, characteristics of underlying malignancy, and additional injuries caused by other treatment modalities such as surgery and chemotherapy [18].

The risk of radiation-induced brain injury depends on the dose (either total dose or per fraction), duration of treatment, and volume of normal brain irradiated [19,20]. All types of radiation therapy and doses were included in our analysis.

Regarding the question related to the possible influence of low doses of peripheral radiation on the effects on the CNS derived from the treatment of other types of tumors, no specific studies on pediatric cancer survivors are reported in the literature. Some works highlight the long-term toxic effect and possible early initiation of senescence on brain cells that could result from exposure to low doses of radiation. However, not being clearly referable to the pediatric population treated for cancer, the authors preferred not to discuss it in the text [21,22].

### 3.1. Neurotoxic Effects of Radiation Therapy

A radiation-induced brain injury is classified into three phases: acute, early delayed, and late delayed toxicities (Table 2) [23].

### 3.2. Neurocognitive Impairment of Radiation Therapy

Pediatric patients have a significant risk for neurocognitive impairment related to brain radiotherapy [25]. Cognitive dysfunction is well described after radiation therapy and is likely related to effects on the brain during the developmental period. Indeed, young age at the time of treatment has been reported as an important risk factor in multiple prospective studies [25,26,27]. Different mechanisms are reported for the radiation-related neurocognitive impairment, including white matter and brain plasticity changes, vascular damage resulting in chronic ischemia, or decreased neurogenesis [28,29].

Cognitive dysfunction generally occurs years after treatment. An annual neuropsychological evaluation is recommended for the early identification of deficits and to initiate educational interventions that are the mainstay of treatment.

Evidence is increasingly emerging that proton beam radiotherapy appears to decrease the incidence and severity of late effects, suggesting that this method may therefore be particularly indicated in the treatment of pediatric tumors. Improved neurocognitive outcomes at present have been demonstrated only in cohorts of pediatric brain tumors [30]. We chose to exclude radiotherapy in primary CNS tumors.

### 3.3. Stroke-like Migraine Attacks after Radiation Therapy

Stroke-like migraine that attacks after radiation therapy (SMART) syndrome is a late and delayed complication of radiation-induced brain injury [31].

The most common symptom is a headache followed by seizures and stroke-like symptoms, such as homonymous hemianopsia, hemiparesis, aphasia, sensory defects, seizures, and migraine-type headaches [32]. The onset of symptoms after radiation treatment is variable; some cases have been reported after 30 years [33,34].

Risk factors are male sex, young age, tumors originating primarily in the central nervous system or metastatic lesions, and radiation dosage more than 50 Gray (Gy) [32,33,35].

Brain MRI findings in SMART syndrome are distinctive and include unilateral hyperintense cortical signal on T2-weighted and FLAIR sequences with gyriform enhancement. The most commonly affected areas are the posterior temporal, parietal, and occipital lobes. The enhancement typically resolves in 2–5 weeks but may persist up to 12 weeks [36].

The pathophysiology of SMART syndrome is likely multifactorial and not well understood due to the rarity of the disease and the lack of histopathological findings in all the reported cases.

The histopathological effects of delayed radiation neurotoxicity are blood-brain-barrier disruption, endothelial cell damage, vascular endothelial growth factor (VEGF) up-regulation, perivascular inflammation, thrombus formation, smooth muscle proliferation, and fibrinoid necrosis of the vessel wall subsequently leading to vessel narrowing and occlusion [32,37].

Despite some patients having an incomplete recovery with permanent imaging sequelae (cortical laminar necrosis) or prolonged unresponsiveness with very slow recovery [36,38], patients with SMART syndrome have a complete recovery in 83% of cases [32].

### 3.4. Acute Late-Onset Encephalopathy after Radiotherapy (ALERT Syndrome)

Acute late-onset encephalopathy after radiotherapy (ALERT syndrome) is a disease entity related to post-irradiation inflammatory endothelial damage or post-radiation mitochondrial damage, as suggested by the similarity of clinical and MRI pattern presentation to stroke-like episodes occurring in inherited mitochondrial disorders [39,40].

Common features include a remote history of irradiation in young and middle age, acute but long-lasting (4–24 days) altered consciousness (Glasgow Coma Scale score 3–10), and clinical improvement after high-dose steroids, associated with multifocal and bilateral brain dysfunction on EEG and MRI.

### 3.5. Secondary Brain Tumors

Radiation may induce secondary brain tumors [41,42]. A high risk for the development of CNS secondary malignancies was reported as a late radiation-induced brain injury. The pathogenic mechanism supposed is direct damage combined with abnormal DNA repair mechanisms related to the use of radiation therapy in combination with alkylating agents and etoposide and is more common in children than in adults [3,43]. Meningiomas and gliomas are secondary brain tumors due to radiation therapy, with a higher risk of radiation-induced damage in younger children and those treated with higher doses of radiation [44].

### 3.6. Radiation-Induced Cerebrovascular Disease

Radiation-induced vasculopathy can occur months to years later after radiation therapy due to head or neck cancer. Focal small-vessel arteriopathy, moyamoya arteriopathy, internal carotid stenosis, and hemorrhage or infarction have been noted in survivors of brain tumors who received radiation therapy several years previously [44,45,46]. The risk factors for developing radiation vasculopathy include patients receiving adjunctive chemotherapy, radiotherapy at a young age, and a higher radiation dose or having other vascular risk factors.

In particular, the development of moyamoya syndrome (MMS) has been rarely reported in children receiving proton beam therapy for brain tumors several years after therapy [47,48]. The risk of developing MMS has been estimated to increase by 7% for every 100 cGy increase in radiation dose above 5000 cGy, with a delay in the occurrence of approximately 5–12 years [49,50].

## 4. CNS Toxicity of Immunotherapy

Summary statement about immunotherapy: unfortunately, this is the sparsest session as it is an innovative therapy with limited data in the childcare literature. We have tried to summarize them to have an overview of the toxicity following this type of treatment. Additionally, in this case, we have organized the toxicities in subsections related to the various types of immunotherapies.

Immunotherapy represents an important new anticancer treatment strategy, particularly antileukemic, in young children and adults. Targeted therapies are needed to treat resistant diseases and to cause less toxicity than chemotherapy in patients with chemosensitive hematologic and oncologic cancer [51].

Immunotherapies have transformed the treatment landscape for multiple solid and hematologic malignancies and confer unique toxicity profiles, which vary depending on the type of immunotherapy, and they are related to the specific mechanism of action [52].

Specifically, central nervous toxicity is described in treatment with Blinatumomab and Chimeric antigen receptor (CAR) T cells, while Rituximab, Ibrutinib, Brentuximab, and Inotuzumab are less associated with central nervous system toxicity. NePlease ensure meaning is retained.urotoxicity frequently represents a common and potentially life-threatening adverse effect from this type of therapy where clinical experience is limited, and grade 3-4 neurotoxicity is a negative prognostic factor for overall survival [53].

### 4.1. CAR T Cell

CAR T cell therapy represents the most advanced T cell therapy to date. CAR T cells are autologous T cells that have been genetically engineered to express the intracellular domain of a T cell receptor fused to the antigen-binding domain of a B-cell receptor [51].

The first clinical trials have shown surprising results, with complete remission (CR) obtained in patients with refractory ALL [52,53]. Presently, CD19 CAR T cells are approved for the treatment of refractory B-cell precursor acute lymphoblastic leukemia (B-ALL) and primary or relapsed diffuse large B-cell lymphomas [54,55]. Once reinfused, CAR T cells attack tumor cells bearing the tumor-specific antigen. Activated T cells produce cytokines and chemokines (IL-2, IFN-gamma, IL-6), usually causing CRS (cytokine release syndrome), the most common side effect [56]. Neurologic toxicity has been reported in clinical trials of CD19 CAR T cells or CAR T cells targeted to non-CD19 antigens. Patients develop expressive aphasia as well as tremor, delirium, and rarely subclinical or clinical seizures and diffuse cerebral edema [57].

Cytokine release syndrome (CRS) and immune effector cell-associated neurotoxicity syndrome (ICANS) are the two most commonly found complications with CAR T cell therapies, which may or may not be associated with the CRS. Early identification is key for treatment [53,58]. Published trials have shown encephalopathy to be the most common symptom with mild cognitive deficits, somnolence, and obtundation; apraxia, tremor, seizures, and cerebral edema were also found [59].

The clinical presentation is heterogeneous and symptoms start approximately 4–5 days after CAR T cell infusion, sometimes after CRS resolution [60]. Usually, all symptoms disappear without sequelae within 10 days [61]. As mentioned, signs and symptoms include headache, tremor, delirium, language disturbance, and seizures but also peripheral neuropathy, weakness, psychiatric and visual disturbances. Acute cerebral edema is rare [62]; it is a “signs and symptoms” based diagnosis. Neuroimaging (MRI or CT scan) shows no significant alteration; a diffuse non-specific generalized slowing appears in electroencephalography. An important finding is the identification of CAR T cells in the cerebrospinal fluid (CSF) in both patients with or without toxicity [63].

The mechanism is still unknown, but recent studies on a large cohort of adult patients with relapsed ALL and treated with CD19-CAR T cells, have related a significant correlation between neurotoxicity and high tumor burden, and specifically, that concerns our review, the correlation between neurotoxicity and a high level of CAR T cells and early increase in serum cytokines [61]. Increased cytokines were also found in CSF, especially IL-6, with high levels considered to be responsible for etiopathogenesis [61].

Specifically, some investigators speculate that an IL-6 receptor blockade with tocilizumab may lead to increased circulating IL-6 in the CNS and can contribute to neurotoxicity exacerbation [55].

In a nonhuman primate model of CD20-targeted CAR T cells, neurotoxicity was associated with the presence of CD20 CAR T cells in CSF and the brain; this model demonstrates that neurotoxicity following CAR T cell infusion is associated with proinflammatory cytokine of CSF [64,65].

Lastly, patients with severe neurotoxicity show endothelial dysfunction and blood-brain barrier disruption [60].

Neurotoxicity treatment is controversial. Corticosteroids, such as dexamethasone 10 mg twice daily associated with tocilizumab, seem to be the most effective [55]. Tocilizumab is an antagonistic IL-6R monoclonal antibody, approved by the FDA for management of CRS treatment after CAR T cell therapy, and is also used for neurotoxicity [55]. The approved dose is 8 mg/kg intravenous (or 12 mg/kg for patients less than 30 kg) and, following the first dose, it may be administered after 8 h. Unfortunately, it does not appear to significantly reduce the incidence or severity of neurotoxicity [65]. Supportive care can be combined; other IL-6-directed therapies, such as siltuximab, or an IL-1 receptor antagonist, like anakinra, are under investigation [66,67].

However, the mechanism of ICANS is unknown, and early identification and prompt intervention are the primary treatment steps. Presently, treatment is based on a multidisciplinary approach, monitoring, supportive care, and corticosteroid and IL6-directed therapy.

### 4.2. Immune Checkpoint Inhibitors (ICIs)

A new recent therapeutic approach in oncology are immune checkpoint inhibitors (ICIs) that target inhibitory checkpoint proteins. These are a component of the immune system that is ensured to modulate T-cell function. Tumor cells have the ability to evade this control mechanism and escape the cytotoxic activity of the lymphocytes themselves [68]. ICIs are also used in some solid and hematological tumors of children (PDL1 inhibitors as Nivolumab, Pembrolizumab, Atezolizumab) and are associated with the risk of adverse side effects mainly affecting the skin, intestine, and endocrine system. [69] However, cases of neurological involvement are reported, mainly represented by encephalitis and aseptic meningitis as well as peripheral neuropathies. To note hypophysitis, which can be associated with ICIs treatments, often presents with concurrent neurologic symptomatology. Moreover, due to the reactivation of T cells caused by ICIs, relapses of pre-existing autoimmune conditions such as multiple sclerosis may occur [70,71].

### 4.3. Blinatumomab

Blinatumomab is a bispecific T-cell engager antibody construct. It contains CD3 and CD19 single-chain variable regions linked by a glycine–serine linker, thus it can bind selectively to CD3 expressing T cells and CD19 expressing B cells, leading to the formation of immune synapses between T cells and B cells [72]. CD19 is expressed in normal and neoplastic B cells, from pre-B cells, until the terminal differentiation in plasma cells [73].

Blinatumomab was initially approved in patients with relapsed/refractory (R/R) non-Hodgkin’s lymphoma (NHL) and in patients with chronic lymphocytic leukemia. Subsequently, in young adults and adults with R/R B-cell precursor ALL and B-ALL, this treatment led to complete remission with minimal residual disease (MRD) [74,75].

Clinically relevant, but fortunately less common, is neurotoxicity attributed to blinatumomab, with global encephalopathy being the most common form, including delirium, seizures, disorientation, and/or cerebral edema [55,76].

If grade 3 or 4 events are less common, lower grade neurological adverse events occur in about half of patients in clinical trials. The mechanism for CAR T cell toxicity is unknown. One possible hypothesized mechanism is the release of neurotoxic cytokines and chemokines by blinatumomab-activated T cells in the central nervous system [77].

The resulting cytokine release leads to local inflammation, disruption of the blood-brain barrier, and neuro-endothelium inflammation [78]. Similar side effects have been reported in patients treated with CD19 CAR T cells [79].

Neurotoxicity represents the most frequent reason for treatment interruptions. The incidence of neurologic events appeared to grow with increasing blinatumomab exposure; however, other factors may also cause this toxicity [80]. Some trials have shown that infusion interruption and steroid treatment are used in treating central nervous system side effects and anticonvulsant drugs can be prescribed if the neurologic effects are seizures [81]. Further investigation is needed.

### 4.4. Brentuximab Vedotin

Brentuximab vedotin (BV), a targeted antibody-drug conjugate (ADC) active against CD30-positive cancer cells, was approved by FDA in 2011 for the treatment of hematologic cancer such as classical Hodgkin lymphoma (HL) and anaplastic large cell lymphoma [82,83].

It is generally a well-tolerated therapy. The main neurological toxicity is peripheral neuropathy, which can limit treatment in more fragile patients; however, it has the most neurological severe side effect (the frequency of grade 3–4 neuropathy is 11%). Other symptoms are headaches and myalgia/myopathy.

Even though peripheral neuropathy is very common and may cause treatment delay or disruptions, a large number of patients have resolution or improvement in neuropathic symptoms after the treatment, as seen in long term follow-up data. Neurological symptoms start days or weeks after 2 to 6 doses of BV administration; treatment is symptomatic and a dose modification can lead to effective treatment [84].

Some trials found possible development of progressive multifocal leukoencephalopathy (PML), a rare opportunistic infection of the central nervous system caused by the John Cunningham virus (JCV), which leads to neurotoxicity not directly linked to therapy. PML mostly manifested in memory loss, hemiparesis, speech dysfunction, gait dysfunction, hemianopsia, and confusion [85].

### 4.5. Rituximab

Rituximab, an anti-CD20 monoclonal antibody, was approved for the treatment of indolent B-cell non-Hodgkin lymphomas in 1997. It was the first therapeutic antibody to be used in oncology. Rituximab has revolutionized the treatment of B-cell malignancies, such as follicular lymphoma (FL), chronic lymphocytic leukemia (CLL), and diffuse large B-cell lymphoma (DLBCL) [86,87]. Several years later, five biosimilars of rituximab have received approval by the EMA with the same therapeutic indications (tositumomab, ibritumomab, ofatumumab, ocrelizumab, and obinutuzumab). Peripheral and central nervous system toxicity are not reported with CD20 monoclonal antibodies. The US Food and Drug Administration (FDA) reported several cases of PML development after treatment with rituximab, such as brentuximab [85]. In 2009, 57 cases were reported, and it was raised to 511 in 2012 [88].

## 5. Conclusions

Neurologic symptoms are common both as primary presentations of malignancy and complications of cancer and its treatment. Given the increasing population of childhood cancer survivors, long-term follow-up and support strategies will be of increasing importance to ensure a high quality of life after childhood cancer.

Despite impressive advances in the field of oncology, the neurological consequences of treatment remain a substantial burden.

CNS complications occur most often during the first months of treatment. Luckily, most are reversible, but there are long-term adverse effects and some can be life-threatening.

Although their incidence is 5–10%, standard recommendations are rare and decisions are usually based on individual considerations. Prevention, early recognition, and treatment of therapy-associated neurotoxicity are imperative to ensure that children can remain on the most effective therapeutic regimens and to improve the neurological function and quality of life of childhood cancer survivors.

## Figures and Tables

**Figure 1:**
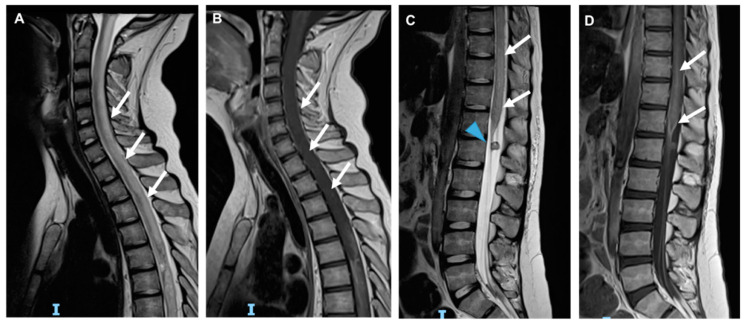
Acute myelitis due to chemotherapy (Nelarabina) in an ALL—T patient. Sagittal MRI shows the abnormal medullar signal of the spine (arrows, **A**) without contrast enhancement after gadolinium administration (arrows, **B**). (**C**,**D**) show an abnormal hyperintensity of the distal part of the medullar signal (arrows, **C**) associated with a peripherical aspecific gadolinium enhancement (arrows, **D**). A metastatic nodule was also seen (arrowhead, **C**).

**Figure 2:**
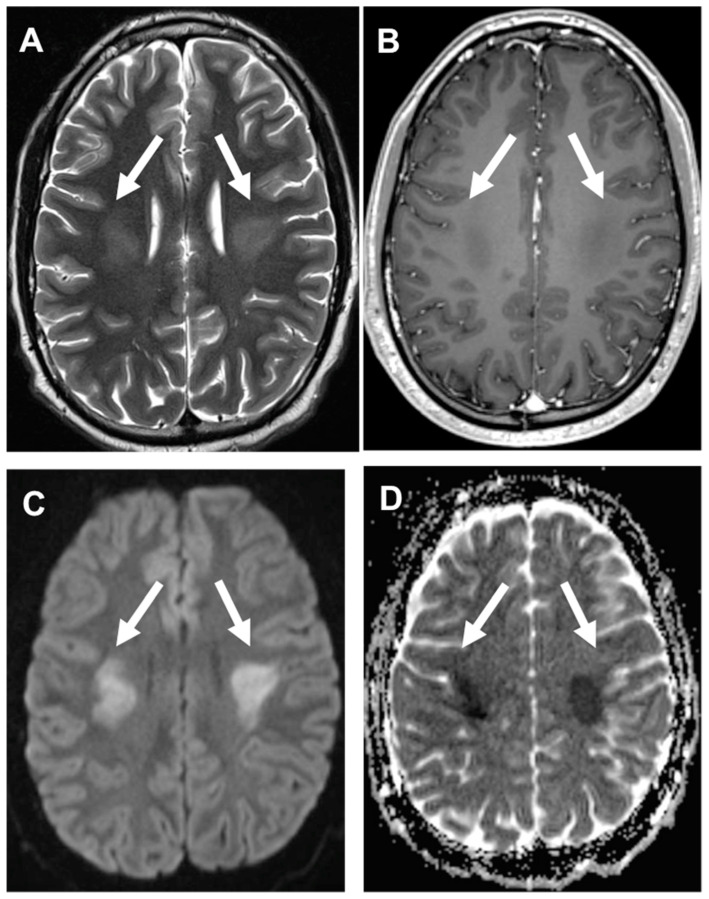
Acute Methotrexate-Induced Neurotoxicity during childhood acute lymphoblastic leukemia (ALL) therapy. MRI shows the characteristic pattern of acute MTX toxicity. Bilateral and symmetric white matter lesion areas of the corona radiata are defined by a high signal on T2 w (arrows, **A**) and no contrast enhancement after gadolinium administration (**B**) associated with restricted diffusion on DWI (**C**) and ADC map (**D**).

**Figure 3:**
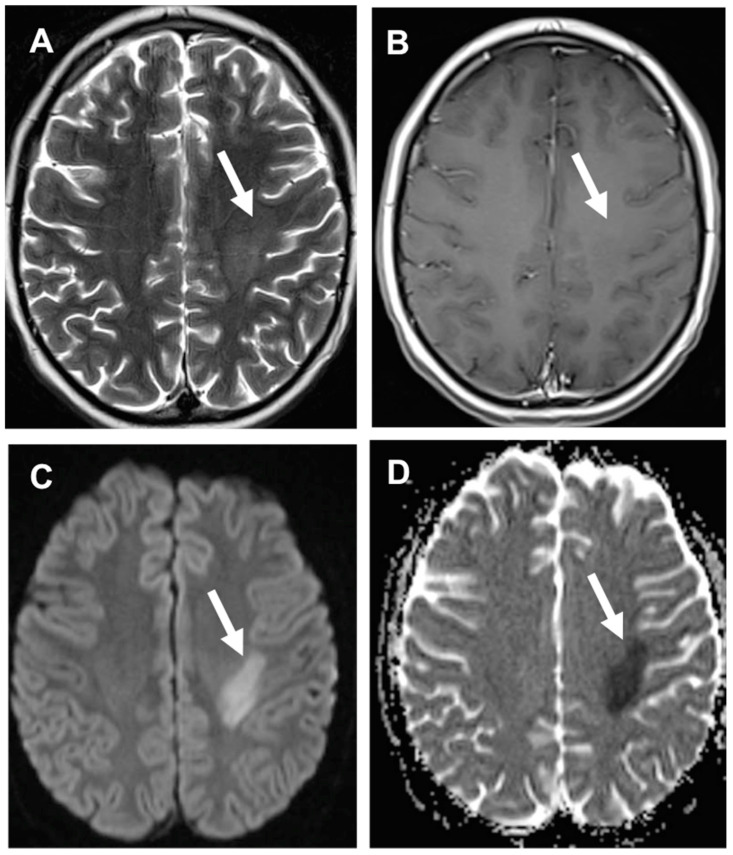
Methotrexate -Induced Neurotoxicity during childhood acute lymphoblastic leukemia (ALL) therapy. Axial T2 w (**A**) shows an asymmetric area of hyperintense signal in the left corona radiata/centrum semiovale (arrow) with restricted diffusion on DWI (**C**) and ADC map (**D**), and no enhancement after gadolinium administration (**B**).

**Figure 4:**
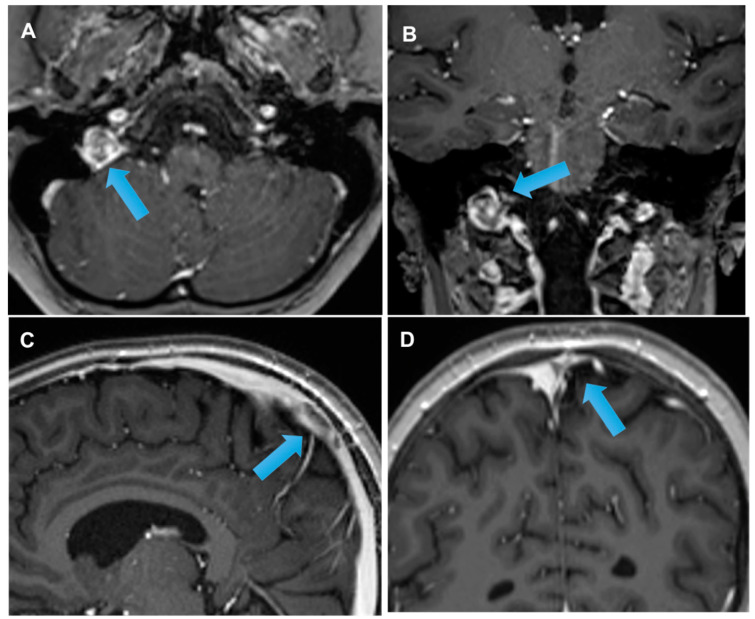
Patient affected by Hodgkin lymphoma during treatment. T1 w MRI after gadolinium administration demonstrates the presence of a complete venous thrombosis of the right jugular vein (arrow, **A**,**B**) and a non-complete thrombus into the superior sagittal sinus on the T1 sagittal view (arrow, **C**) and coronal view (arrow, **D**).

**Table 1:** Most frequently used chemotherapy drugs in pediatric onco-hematology and their CNS effects *.

DRUG	NEUROLOGIC SYMPTOMS
***ALKYLATING AGENTS***	
***Cyclophosphamide***	− Reversible encephalopathy: dizziness, blurred vision, and confusion (uncommon). − Reversible cerebral vasoconstriction (rare) [1,4,5]
***Ifosfamide***	− Encephalopathy: from mild confusion or somnolence to more severe forms with hallucinations, psychosis, disorientation, memory loss, seizure, delirium, and rarely, coma and death. Incontinence, muscle twitching, focal motor deficits, facial nerve palsy, aphasia, mutism, and myoclonus are also common clinical symptoms [1,4,6]. Occurs in 10–30% of pediatric patients [6], has nonspecific CT and MRI features, but may have EEG triphasic waves (very common) [1,7].
***Busulfan***	− Headaches (common) − Dizziness (uncommon) − Generalized tonic-clonic seizures: after 3–4 days of administration, anticonvulsant prophylaxis is recommended (uncommon) [1,4,6].
***Platinum compounds (cisplatin, carboplatin, and oxaliplatin)***	− Sensory ganglionopathy (rare) − muscle weakness/acute musculoskeletal pain (rare) − SIADH (very common) − Cranial neuropathies: ototoxicity, ageusia (uncommon) − Ataxia (uncommon) − Lhermitte’s phenomenon (rare) − Urinary retention (rare) − Confusional states, seizures, stroke, chronic leukoencephalopathy, ocular toxicity, and diffuse encephalopathy are mainly associated with cisplatin (rare) [1,5].
***ANTIMETABOLITES***	
***Cytarabine***	− Pancerebellar syndrome: dysarthria, dysmetria, ataxia, nystagmus, and dysdiadochokinesia, due to direct damage of Purkinje cells (common) [1,4,6]. − Seizures and confusional syndromes: after intrathecal injection (common) [8]. − Myelitis/cauda equine syndrome and acute chemical meningitis: after intrathecal injection especially when no prophylactic dexamethasone is administered [4] (Figure 1).
***Fludarabine***	− Deterioration of vision: with photophobia, optic neuritis, and cortical blindness (common). − Seizures (common) − Ataxia (common) − Tremor (common) − Myelopathy [1,4] (common) − Leukoencephalopathy: high dose-related; presented with paralysis, pyramidal tract dysfunction, altered mental status, hallucinations, seizure, or extremely severe forms with several deficiencies worsening progressively to coma and death even months after exposure (rare) [4].
***Methotrexate***	− Chemical meningitis: fever, headache, nausea or vomiting, stiff neck, lethargy, generally self-limited occurring in about 5–40% of patients (very rare). − Leukoencephalopathy: stroke-like episodes with transient hemiparesis, speech impairment, dysphagia, diplopia, hemi-sensory deficit, and seizures. In the case of asymptomatic patients, particular MRI features such as white matter T2/FLAIR hyperintensities and diffusion restriction may configure a peculiar asymptomatic leukoencephalopathy [1,6] that can precede the appearance of clinical symptoms [9,10] (very rare) (Figure 2). − Adhesive arachnoiditis: compression of nerve roots and their blood supply (very rare) [4]. − Transverse myelopathy: low back or leg pain followed by paraplegia and sensory loss, flaccid paresis, and fecal and urinary incontinence/retention, depending upon non-inflammatory vacuole demyelination and necrosis of the spinal cord (very rare) [4] (Figure 3).
***NATURAL PRODUCTS***	
***Asparaginase***	− Cerebral sinovenous thrombosis (common) [1,4,6] − Encephalopathy (rare) − Seizures (rare) − Coma (rare) − Focal deficits (rare) − Headache (uncommon) [1]
***IMMUNOSUPPRESSANTS***	
***Cyclosporine A (CSA)***	− Metabolic encephalopathies: exacerbated by hypomagnesemia, hypertension, acute kidney injury, hypocholesterolemia, and corticosteroids [9,10]. Characterized clinically by paresthesia, headache, seizures, confusion, visual hallucinations, cortical blindness, ocular flutter, cerebellar-like syndromes, leukoencephalopathy, encephalopathy, and intractable epilepsy associated with mesial temporal sclerosis (common) [11]. − Irreversible CSA leukoencephalopathy (uncommon) [12] − PRES (Posterior reversible encephalopathy syndrome): typical symptoms are epileptic seizures, headache, alteration of the visual system and mental status, and cerebral hemorrhage (common) [13].
***Tacrolimus***	− Vascular endothelial damage: seizures, headache, nausea, altered mental status, confusion, verbal disorder, cortical blindness, and hemiplegia (common). − Encephalopathy (uncommon) [14] (Figure 1 and Figure 4)
***OTHERS***	
***Dimethyl sulfoxide (DMSO)***	− (Used in the conservation process of stem cells in autologous transplantation) − Stroke (uncommon) − Seizures (uncommon) − PRES (uncommon) − Transient amnesia (uncommon) − Blindness (uncommon) − Reversible cerebral vasoconstriction syndrome (uncommon) [11]

* Incidence estimated according to classification approved by World Health Organization.

**Table 2:** Signs and symptoms due to radiotherapy toxicity subdivided according to time of onset.

ACUTE (Few Days after Radiation)	EARLY DELAYED (Weeks/Months after Radiation)	LATE DELAYED (Several Months/Years after Radiation)
Neurologic changes, cerebral edema, seizures, altered level of consciousness, persistent headache, hemiplegic symptoms, hallucinations, and visual disturbances [24,25]	Radiation somnolence syndrome (prolonged periods of sleep, irritability, fever, nausea, vomiting, cerebellar ataxia, anorexia, dysphagia and dysarthria, and headaches) [3]	Vascular abnormalities, demyelination [25,26]

## Abbreviations

ADEM	Acute disseminated encephalomyelitis
CNS	Central nervous system
CSA	Cyclosporine
Ara-C	Cytosine arabinoside
GVHD	Graft versus host disease
HSCT	Hematopoietic stem cell transplantation
MTX	Methotrexate
PRES	Posterior reversible encephalopathy syndrome
